# A 3D Human-Machine Integrated Design and Analysis Framework for Squat Exercises with a Smith Machine

**DOI:** 10.3390/s17020299

**Published:** 2017-02-06

**Authors:** Haerin Lee, Moonki Jung, Ki-Kwang Lee, Sang Hun Lee

**Affiliations:** 1 Intelligence and Interaction Lab., Graduate School of Automotive Engineering, Kookmin University, 77 Jeongneung-ro, Seongbuk-gu, Seoul 02707, Korea; lhr2010@naver.com; 2 AnyBody Technology A/S, Niels Jernes Vej 10, Aalborg East 9220, Denmark; mj@anybodytech.com; 3 Biomechanics Lab, Department of Sports Science, Kookmin University, 77 Jeongneung-ro, Seongbuk-gu, Seoul 02707, Korea; kklee@kookmin.ac.kr

**Keywords:** squat, biomechanical analysis, musculoskeletal model, Gaussian process regression, motion generation, digital human modeling

## Abstract

In this paper, we propose a three-dimensional design and evaluation framework and process based on a probabilistic-based motion synthesis algorithm and biomechanical analysis system for the design of the Smith machine and squat training programs. Moreover, we implemented a prototype system to validate the proposed framework. The framework consists of an integrated human–machine–environment model as well as a squat motion synthesis system and biomechanical analysis system. In the design and evaluation process, we created an integrated model in which interactions between a human body and machine or the ground are modeled as joints with constraints at contact points. Next, we generated Smith squat motion using the motion synthesis program based on a Gaussian process regression algorithm with a set of given values for independent variables. Then, using the biomechanical analysis system, we simulated joint moments and muscle activities from the input of the integrated model and squat motion. We validated the model and algorithm through physical experiments measuring the electromyography (EMG) signals, ground forces, and squat motions as well as through a biomechanical simulation of muscle forces. The proposed approach enables the incorporation of biomechanics in the design process and reduces the need for physical experiments and prototypes in the development of training programs and new Smith machines.

## 1. Introduction

The barbell squat is a fundamental physical exercise for strengthening the lower body and core muscles. It is an integral part of training and conditioning programs in sports, rehabilitation, and fitness. The free barbell squat requires a degree of balance and coordination during motion, and the knee and lower back experience greater forces and torques than those to which they are accustomed [1]. There has been much research on the biomechanical analysis of the free squat with a particular focus on muscle activity [2], safety of knee structures (ligaments, menisci, and cartilage) [3], and different squat techniques according to the amount of knee flexion, stance width, foot angle position [4,5], external load type and positioning [6], and speed of execution and external load intensity [7]. As an alternative form of the exercise, devices that move on linear tracks have been developed. One such device is the Smith machine, in which a barbell is horizontally constrained to move up and down while sliding along vertical steel tracks. The machine allows variation in the anterior–posterior foot placement and bar slope as well as external load and squat depth. Therefore, the Smith squat offers a wider range of exercise positions than the free squat and concurrently a wider range of possibilities for modulating the distribution of muscle activity and joint loads [8]. For the Smith squat, several researchers have focused on testing issues, including muscle force, peak lifting velocity, maximum power [9,10,11], various training effects on other sports [12,13,14], and joint torques and loads [2,15]. Two-dimensional (2D) biomechanical models for the Smith squat have been developed to evaluate knee and hip torques at various foot positions [2,15] and tibiofemoral joint loads as a function of external load, trunk tilt, and body configuration [2].

2D musculoskeletal models have been developed based on the assumption that there are no bilateral differences in the forces and moments generating squat motions. However, it was reported that there were significant bilateral differences in the ground reaction forces and joint torques depending on loading conditions [16,17]. Flanagan et al. [16] have observed that the average vertical ground reaction force of the left side has a slightly (~6%) larger magnitude than the right side. The average net joint moments at the left and right hip, knee, and ankle joints during the barbell squat to differ by an average of ~16%, ~14% and ~20%, respectively. Asymmetrical movement patterns such as this could theoretically lead to injury as it results uneven distribution of forces. Therefore, it is necessary to develop a 3D musculoskeletal model to analyze asymmetrical squat motions.

In recent years, digital human modeling has been a fast growing area that bridges computer-aided engineering, design, human factors, applied ergonomics, and sports coaching and training and plays an important role in product design, prototyping, manufacturing, sports biomechanics, and many other areas [18,19,20]. A digital human model (DHM) is a digital representation of a human in three-dimensional (3D) space that can be moved and manipulated to simulate real and accurate movements of people. Digital human modeling is a process of developing DHMs using an anthropometric and biomechanical database for predicting performance and/or safety in a virtual environment. This enables the reduction or elimination of the need for physical prototypes in new product design and the earlier incorporation of ergonomic science in the design process. DHMs can be classified into three groups: digital mannequins such as Ramsis, Jack, and Safework [21], deformable finite element models such as HUMOS and H-model [22] and musculoskeletal models such as OpenSIM [23], LifeModeler [24], and AnyBody [25]. Musculoskeletal models are widely used in sports biomechanics for simulating torques and loads of joints and muscles [26,27]. 

The DHM technique has previously been partially applied to analyze the Smith squat exercise. That is, a 3D musculoskeletal modeling and simulation of the Smith squat have been performed using commercial or open source packages. NASA has developed OpenSim models to simulate lower-body resistance exercises on some devices that allow astronauts to perform resistance exercise on the International Space Station, on which muscle mass is lost owing to reduced gravity [28,29]. However, the models were basically 2D ones because the motion of the human-machine system was defined by prescribing the sagittal plane motion of the ankle, knee, hip and back joints. Kim et al. [30] investigated and compared the electromyography (EMG) data and muscle forces during free and Smith squats through physical experiments and biomechanical analysis using AnyBody while varying the guide bar angle. On the basis of the analysis results, they proposed new design for the Smith machine in which two guide bars of a barbell bar are tilted by 10.7°. However, they used free squat motion data to simulate the Smith squat, which may cause large errors in analysis results. This model is also a 2D one because the kinematic structure of the system was modeled in the sagittal plane. Therefore, it is necessary to develop a reasonable squat motion synthesis method and a 3D DHM integrated with the machine to investigate the effects of training programs or equipment design with varying input parameters.

Significant research has been performed for the simulation of human motions for computer-aided ergonomic design [31]. A multilayer perceptron neural network was trained to generate the arm movements of a virtual mannequin based on the kinematic database of a participant [32]. Optimization approaches that minimize energy or muscular efforts have been developed to simulate upper body [33] and full-body motions [19,34]. Inverse kinematic methods from robotics have also been utilized to predict upper body reach motion [35,36]. Other recent approaches for motion simulation have utilized a database of motions for motion modeling and prediction [37,38] or combined existing motions to generate new ones [39]. Several approaches have used statistical analysis (e.g., regression) of motion-captured data to form predictive models for a sequence of postures [40,41]. A regression model was fitted from a large set of reach motion data to predict average joint angle–time trajectories and corresponding angle–time confidence envelopes [42]. In addition, to make end effectors arrive at intended target locations, the final postures of predicted motions were rectified using an inverse kinematic method [43]. Recently, probabilistic-based methods have been used to create new motions for different applications such as motion editing [44] and a style-based inverse kinematic system [45,46]. In particular, a generative model based on the Gaussian process regression (GPR) can directly learn from the training data without extracting any interpolation parameter. It defines a probability density function over new motions, which can be used to predict missing frames. It works well with a small data set and gives good results in predicting animations and kinematic configurations [43,44,45,46,47].

In the present study, we applied a digital human modeling technique to the Smith squat exercise. We developed a digital human–machine-integrated model and probabilistic-based motion synthesis algorithm for a 3D biomechanical analysis of symmetric and asymmetric Smith squat motion. To validate the human–machine model, EMG, external forces, and squat motions were captured through physical experiments using varying independent variables such as the foot placement and slope of barbell bar guides. A probabilistic-based motion synthesis system was developed using GPR. The analysis results, including joint torques and muscle activities, are useful for designing training programs and the Smith machine. The proposed approach is expected to enable the incorporation of biomechanics in the design process and reduce the need for physical experiments and prototypes in the development of training programs and new Smith machines.

This paper is organized as follows: Section 1 introduces related work as well as the research background and objectives. Section 2 describes variables, apparatus, and procedures required for the Smith squat experiment. Section 3 presents a method for constructing and validating an integrated human–machine–environment model. Section 4 describes the construction and validation of the squat motion synthesis system based on the GPR algorithm. Section 5 compares the analysis results of synthesized and captured motions. Section 6 presents and discusses the analysis results of the Smith squat exercise with varying input values. Section 7 concludes this work and suggests future work.

## 2. Experimental Method

### 2.1. Independent Variables

**Figure 1:**
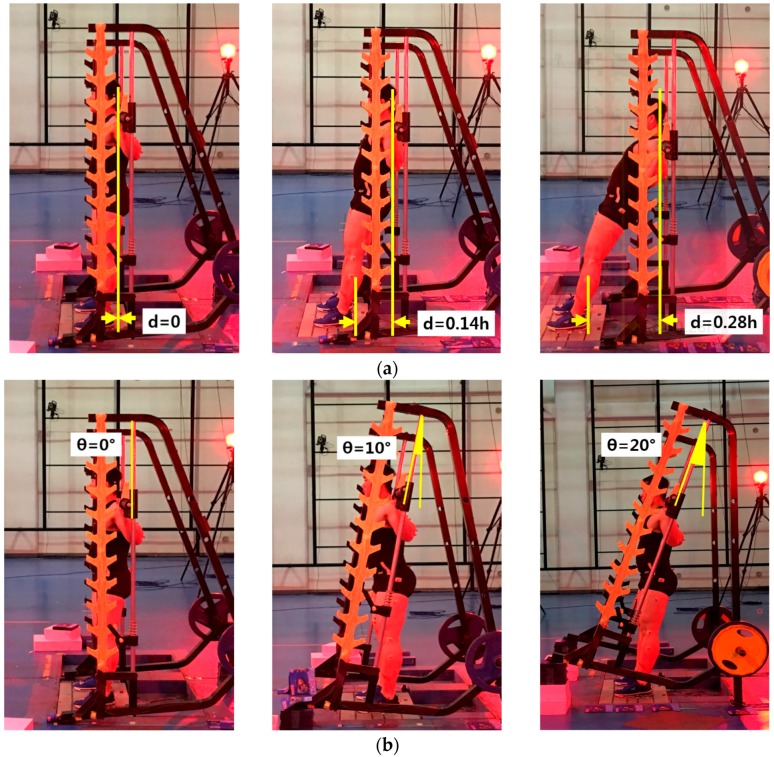
Independent variables of experiment: (**a**) foot position and (**b**) angle of barbell bar guides.

For the Smith squat exercise, there are many possible independent variables such as the foot placement, slope of barbell bar guide, stance width, foot angle position, external load, execution speed, user’s age, user’s sex, and anthropometric parameters. In this study, we selected two dominant independent variables for the Smith squat exercise, i.e., the foot position and slope of the barbell bar guide. The foot position, denoted as D, is defined as the perpendicular distance from the center of the bar during the initial pose, as shown in Figure 1a. Three foot positions were used. At the most posterior position, the ankle was placed directly under the bar during the initial pose. At the most anterior position, the upper leg was almost parallel to the floor. The remaining position is at the middle of the two extreme positions. Three positions are represented as the ratios of the total height of the user (h), which are 0, 0.14 h, and 0.28 h. For instance, if a participant’s height is 1800 mm, foot positions were 0, 250, and 500 mm from the barbell bar center during the initial pose. Guide angles were selected as 0°, 10°, and 20°, as shown in Figure 1b.

The participants performed back squats with their thighs parallel to the floor in the bottom position (i.e., parallel squatting). Their feet were planted straight at the shoulder width (narrow stance width). External load on the shoulder was 267 N.

### 2.2. Apparatus

**Figure 2:**
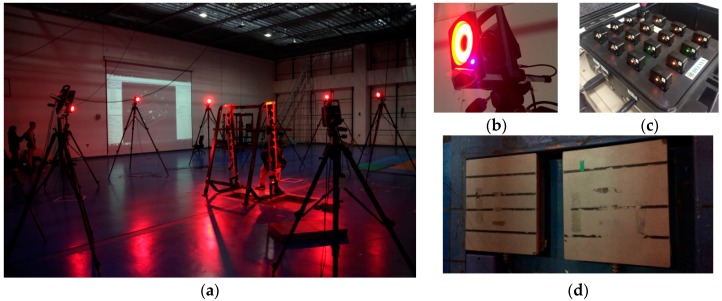
Experimental apparatus: (**a**) motion capture studio at the Advanced Institute of Convergence Technology; (**b**) Vicon T-120 camera; (**c**) Delsys Trigno wireless EMG sensors; and (**d**) AMTI OR6-7 force plate.

To capture Smith squat motions, as shown in Figure 2, we used an optical motion capture system equipped with 12 T160 infrared cameras (Vicon, Oxford, UK) whose maximum frame rate at full resolution was 120 fps [48]. We attached 39 markers to the participant according to the positions of the Vicon Plug-in-Gait marker set [49]. The motion capture data were converted and exported by Nexus to the C3D data file format for AnyBody to import as an input motion data file.

To validate boundary conditions for biomechanical analysis, two OR6-7 force plates (AMTI, Watertown, MA, USA) [50] were placed on the ground to measure the reaction force (maximum 4413 N) against the sole of the feet. The measurement device crosstalk was less than 2% among all channels, and Fx, Fy, and Fz hystereses and nonlinearity were ±0.2% of the full-scale output.

To measure and record surface EMG activity during ingress/egress movements, we used a Trigno wireless EMG instrument (Delsys, Natick, MA, USA) as shown in Figure 2 [51], completely integrated with the motion capture system. Each EMG sensor has a built-in tri-axial accelerometer, transmission range of 40 m, and rechargeable battery. In this study, six wireless EMG sensors were attached to the lower body skin, i.e., the vastus medialis, vastus lateralis, and rectus femoris of both legs. Forces activated by these muscles were simulated using a biomechanical analysis system and then compared with the EMG measurement data to validate analysis results.

### 2.3. Procedure

Total 14 participants were selected from the student population of Kookmin University after detailed screening for musculoskeletal disorders. They were 24 (SD = 1.9 years) years old, 1770 mm (SD = 82.6 mm) in height, and 78 kg (SD = 11.6 kg) in weight. They have performed weight training for the last one year. They were informed about the goal of the study and experimental methods and were asked to fill consent forms and a basic questionnaire. Before performing experiments, they undertook preparation exercises to prevent injuries and practiced squatting using the Smith machine to adapt to the experimental environment.

**Figure 3:**
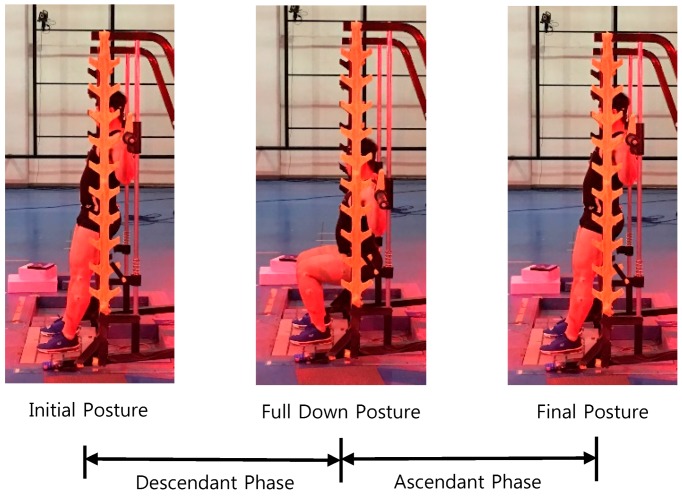
Squat motion cycle.

As shown in Figure 3, a cycle of squat motion was divided into descending and ascending phases by the initial, full-down, and final postures. In the full-down posture, their thighs were parallel to the floor. External load to the shoulder was 267 N. A squat cycle was executed in approximately 6 s, which was controlled using a metronome. As presented in Table 1, squat motions in the nine cases marked by the circular symbol were measured using EMG measurements. For each case, squat motions were measured five times. To validate newly generated motions, Smith squats were also measured five times for each case, marked by the triangular symbol.

## 3. Human–Machine–Environment Integrated Model

### 3.1. Musculoskeletal Model

In this study, biomechanical analyses of squat movements were performed using AnyBody. A musculoskeletal model for the participant’s body was created, markers were attached to the model, and boundary conditions were specified at contact positions with the machine and ground. Then, using AnyBody, joint loads and moments and eventually muscle forces for the given input data were determined. The musculoskeletal model was constructed using a full-body model stored in the AnyBody Managed Model Repository (AMMR) v.1.6.3. The model consisted of 25 segments, 16 joints, and 804 muscles. Each arm and each leg have 140 and 159 muscles respectively while the truck and head parts have 206 muscles. Here, the joints were the neck, sternoclavicular, glenohumeral, elbow, wrist, pelvis–thorax, hip, knee, and ankle joints. The model was configured to match the body dimensions of the participant, and the default values of AnyBody were applied to the material properties of the body segments, joints, and muscles. The maximum stress on a muscle was 9.0 × 10^5^ Pa (=90 N/cm^2^), and the maximum contraction force for each muscle was obtained by multiplying the cross-sectional area of that muscle by the maximum stress.

**Figure 4:**
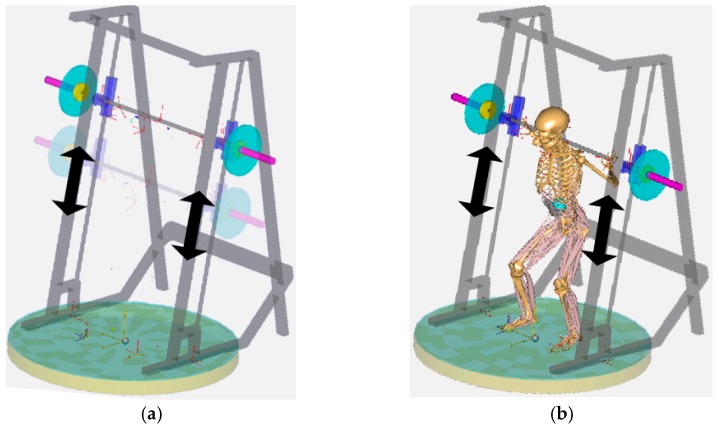
Human–machine-integrated model in AnyBody: (**a**) solid model for Smith machine; and (**b**) human–machine-integrated model.

Solid models were created for the frame, a barbell, and bar holders of the Smith machine using SolidWorks. The models were imported into AnyBody, as shown in Figure 4. Mating conditions were applied to assemble the parts. The barbell linearly moves along the guide bar.

**Figure 5:**
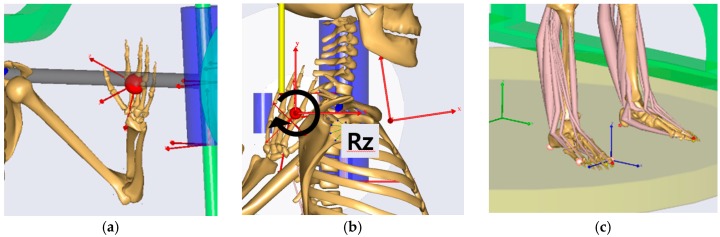
Modeling of interaction among a human, machine, and environment: (**a**) weld joints to fix hands on barbell; (**b**) revolute joints to model contact of barbell bar with shoulder area; and (**c**) weld joints to fix feet on ground.

The human body contacts the Smith machine and ground. Their interactions were modeled using joints such as spherical (three degrees-of-freedom (DOFs)), cylindrical (one DOF), revolute (one DOF), prismatic (one DOF), universal (two DOFs), trans-spherical (four DOFs), and weld (zero DOF) joints in AnyBody. Hands were fixed on the barbell bar using weld joints between virtual points on the bar and hands, as shown in Figure 5a. The barbell bar contacts the shoulder area, and their interaction was modeled using revolute joints between the virtual points of the bar and trunk which are on the axis of the bar to allow the rotation of the trunk with respect to the bar, as shown in Figure 5b. Feet were fixed on the ground using standard joints, as shown in Figure 5c.

For the human model, the 16 joints had 38 DOFs and the pelvis had six DOFs that consisted of three translation components and three rotation angles. The barbell had just one movement capability, i.e., translation along the bar guide. Therefore, the total DOFs of the system was 45 with the single DOF of the barbell and 44 DOFs of the entire body model in space. Squat motion was derived using the joint angle data for the flexions/extensions of the pelvis–thorax, hip, and knee joints, just as that derived for a 2D rigid body model [2,15]. Other joint angles were determined from constraints among the human body, machine, and environment or were set at constant values. All rotations of the neck and sternoclavicular joints and the flexion/extension of the glenohumeral joint were removed by setting constant values for the initial posture. All rotations of the wrists and elbows, external/internal rotation and abduction/adduction of the glenohumeral joint and the lateral bending and vertical rotation of the pelvis–thorax joint were removed using constraints between the barbell bar and body. The abduction/adduction and external/internal rotation of the hip, plantarflexion/dorsiflexion and abduction/adduction of the ankle, and translations and rotations of the pelvis were eliminated using constraints between the feet and ground as well as the shoulder and bar. The flexions/extensions of the pelvis–thorax, hip, and knee joints were specified according to the values of captured or generated motions.

In the simulation environment, ground reaction forces (GRFs) cannot be measured from force plates. Fortunately, AnyBody has the capability to predict GRFs using artificial muscles, which are usually referred to as a conditional contact [52,53]. Using the equations of motion and a scaled musculoskeletal model, GRFs and moments were derived from 3D full-body motion. The method was implemented in AnyBody and utilized in this study.

### 3.2. Validation of Model Using Measured Data of Ground Reaction Forces and EMG

A simulation was performed via AnyBody using the human body model, contact conditions, and squat motion data. As a result, the angle, angular velocity, angular acceleration, and torque for each DOF of a joint and the contraction force of each muscle and GRFs during movement were calculated. To evaluate the similarity between the measured and calculated data, we adopted the normalized root mean square error (NRMSE) and the Pearson correlation coefficient denoted by r as the measures of accuracy. Here, NRMSE is defined as RMSE divided by the range of the data and expressed as a percentage.

**Figure 6:**
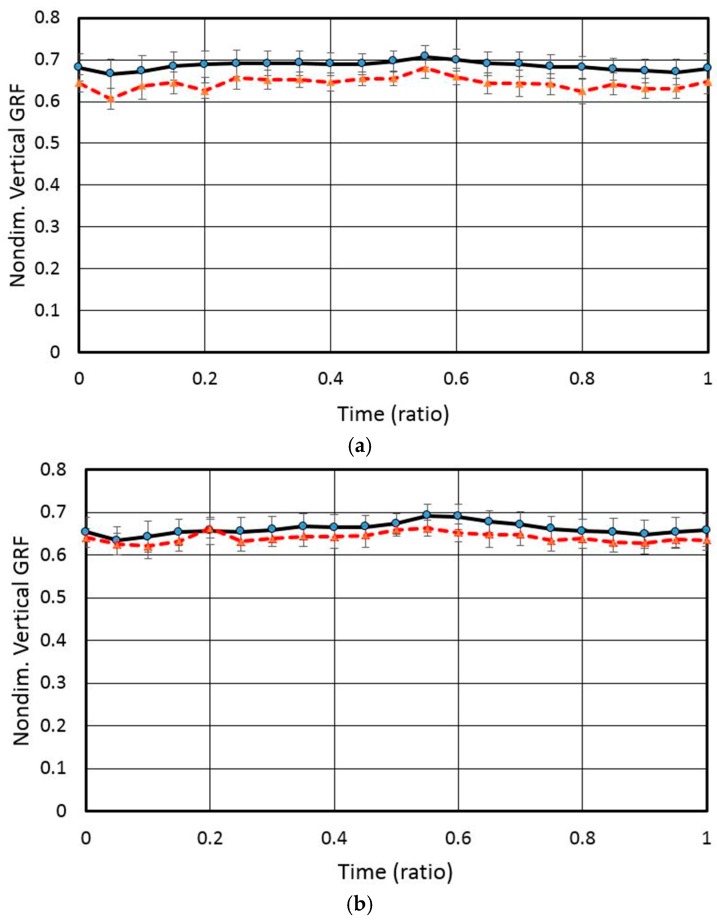
Comparison of measured average normalized vertical GRF with simulated results for the case where the foot position was 0 mm and guide angle was 0° from barbell bar center: (**a**) left vertical GRF and (**b**) right vertical GRF.

To validate GRFs on the feet, we performed a comparison between reaction forces measured for all participants in the experiment and corresponding external forces simulated using AnyBody. The forces for each participant were by dividing by his/her weight to eliminate the effect of the weight. Figure 6 illustrates the average nondimensional vertical reaction force of the feet in the case where the foot position was 0 mm and guide angle was 0° from the barbell bar center during squat motion. The dotted line depicts the average force simulated using AnyBody, and the solid line depicts the average reaction force measured in the experiment. The error bars represent 95% confident intervals. Simulated and actual exerted forces exhibited very similar tendencies and magnitudes, which demonstrates that the GRF prediction method implemented in AnyBody can be considered feasible. The NRMSEs between the measured and simulated GRFs on the left and right feet are 32.90% and 46.6%, respectively. The Pearson correlation coefficients for the left and right feet are −0.44 (*p* = 0.045) and 0.781 (*p* = 0.000), respectively, which mean there is no significant linear relationship between the measured and simulated GRFs for the left foot.

**Figure 7:**
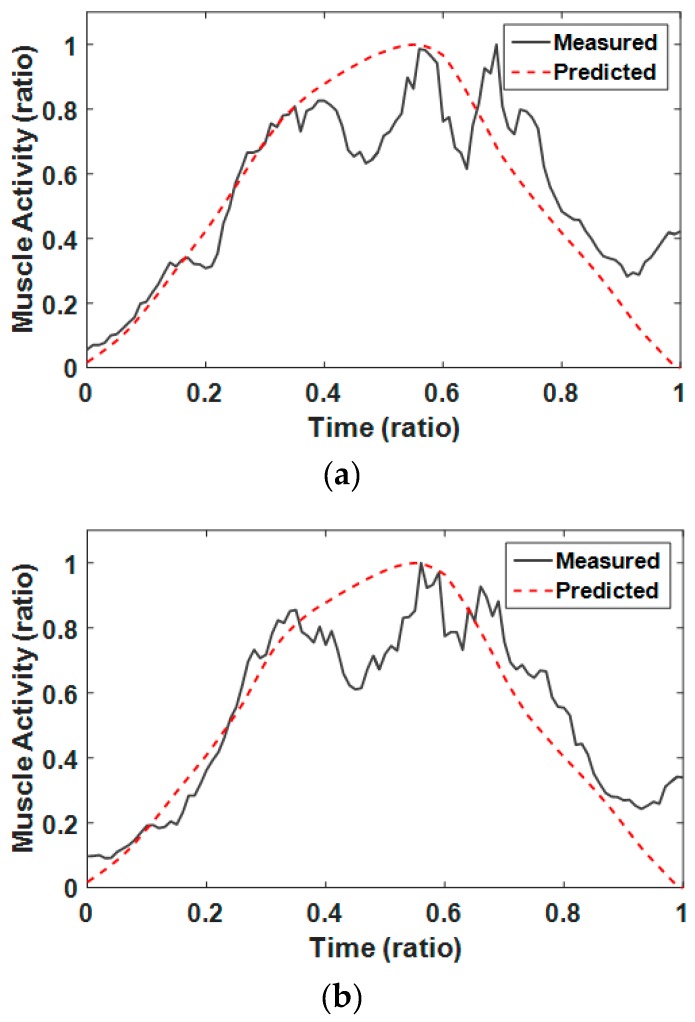

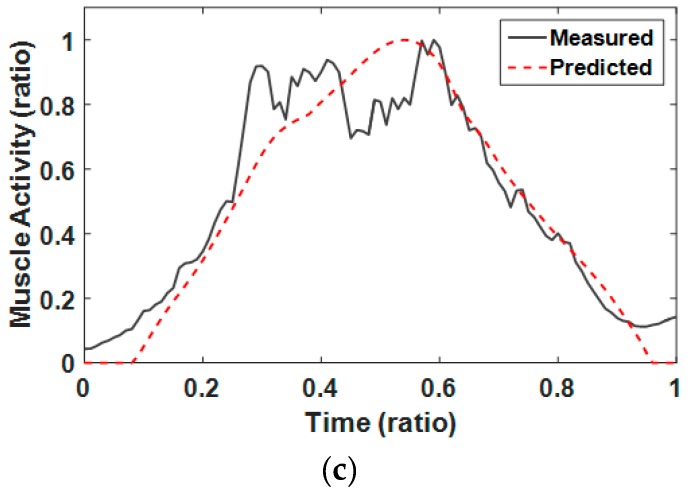
Comparison of measured EMG data of right leg with simulated muscle activities in the case where the subject was 1800 mm in height and the foot position and guide angle were 250 mm (=0.14 h) and 0° respectively: (**a**) vastus lateralis; (**b**) vastus medialis; and (**c**) rectus femoris.

To validate our analysis model for Smith squats, we analyzed muscle activities of the subject who was selected for the GRF validation. Figure 7 shows muscle activities measured and simulated for the vastus lateralis, vastus medialis, and rectus femoris of the right leg for the case where the foot position was 250 mm (=0.14 h) and guide angle was 0° from the barbell bar center. Simulated and measured values were normalized by their maximum values to facilitate pattern comparison. As shown in Figure 7, the patterns of simulated muscle activities are well matched to those of EMG data. As shown in Table 2, the average NRMSEs of the vastus lateralis, vastus medialis, and rectus femoris of the right leg were 22.1%, 15.1%, and 13.8%, respectively. The total average of the NRMSEs for all three muscles was 17.0%. These numbers show that simulated muscle activities can predict real motions quite well. Table 3 shows the correlation analysis results between simulated muscle activities and EMG values for three representative muscles of the right leg for six cases. The average Pearson correlation coefficients of the vastus lateralis, vastus medialis, and rectus femoris of the right leg were 0.820, 0.860, and 0.897, respectively. The total average of the coefficient for all three muscles was 0.859. The high positive correlation coefficients show that the analysis model is valid for predicting muscle activities. In addition to the NRMSEs and the correlation coefficients, we introduced two more measures to compare the measured and simulated muscle activities, which are the peak timing for the maximum muscle activities and the total muscle activity. Total muscle activity is defined as the summation of all muscle activities by excluding below 20% muscle activity values [54]. Table 4 shows the peak timing for the maximum muscle activities in the measured EMG and the calculated musculoskeletal model results. Table 5 summarized the total muscle activities during a Squat exercise cycle in each case.

## 4. Motion Synthesis

### 4.1. Motion Capture Training Data

**Figure 8:**
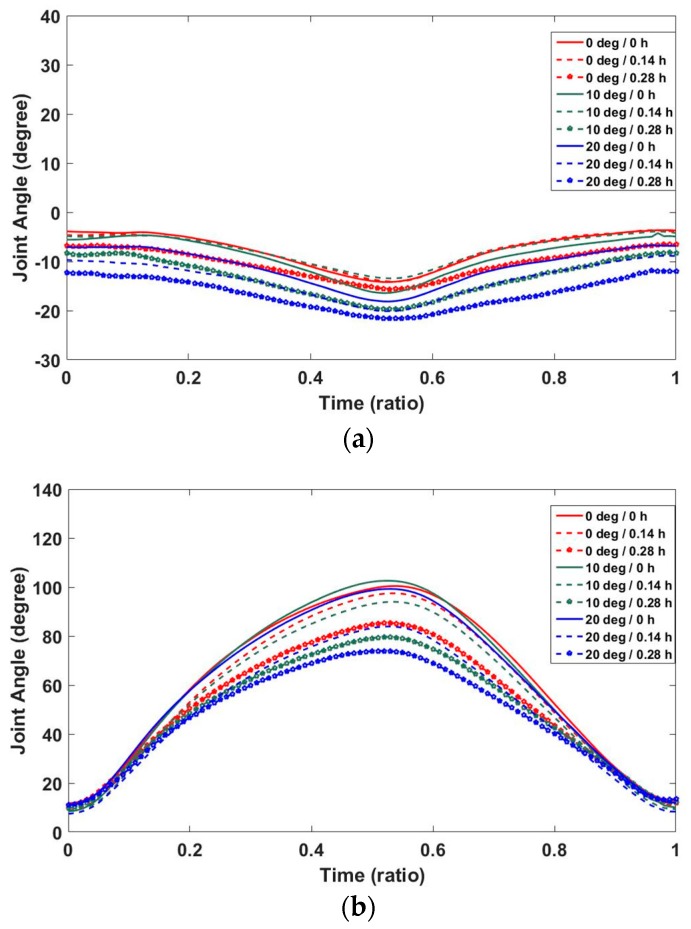

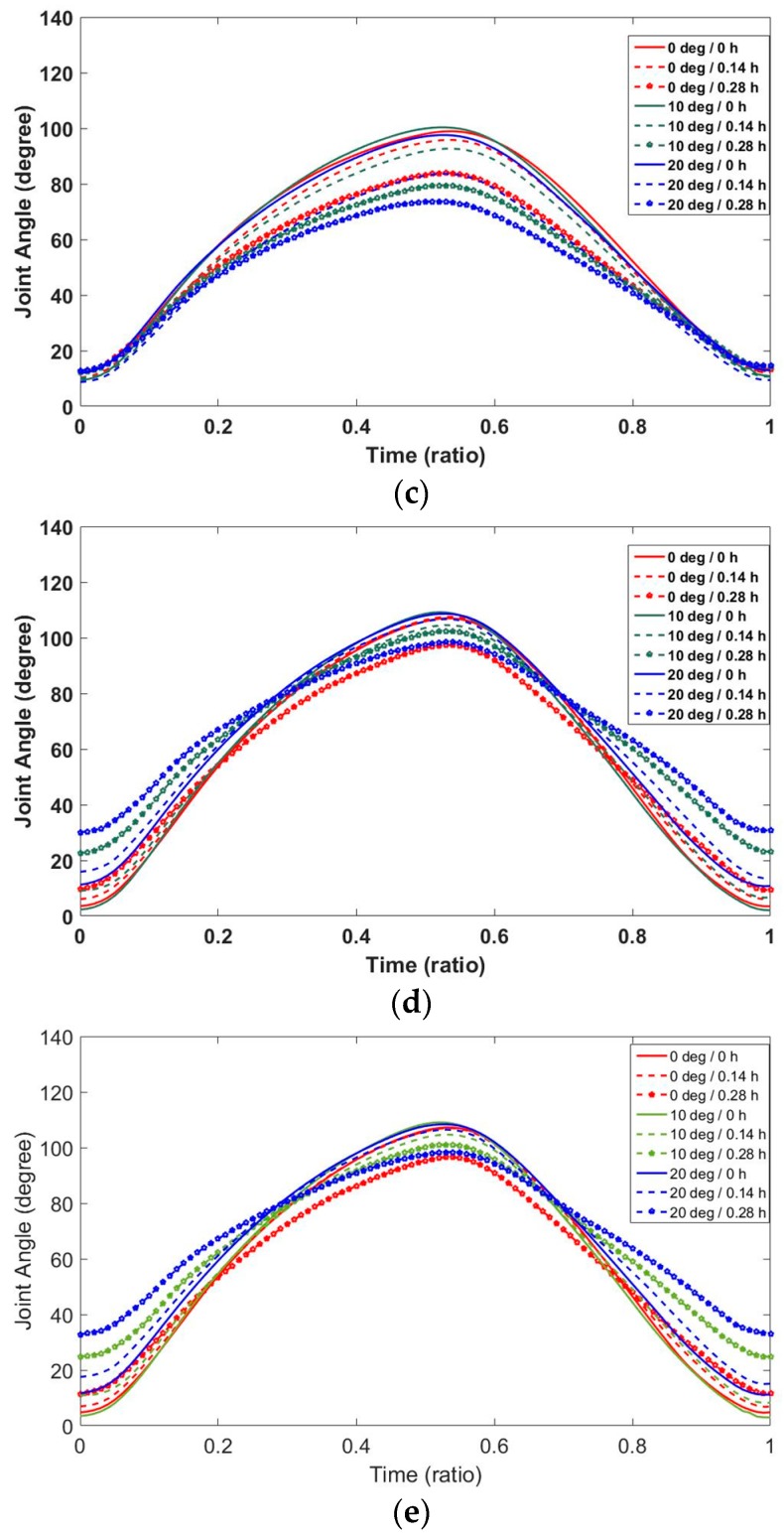
Flexion/extension of pelvis–thorax, hip, and knee joints for nine cases: flexions/extensions of (**a**) pelvis–thorax joint; (**b**) left hip joint; (**c**) right hip joint; (**d**) left knee; and (**e**) right knee.

The model was driven by five angles for the flexion/extension of the pelvis–thorax, two hip, and two knee joints. The database had 630 motion capture datasets measured for 14 participants and nine cases repeated five times each. For each joint angle, average curves for the nine cases are shown in Figure 8. The data were used for training our motion synthesis algorithm based on GPR to generate motion for arbitrary values of two independent variables.

### 4.2. Motion Synthesis Method Using Gaussian Process Regression

Here, we briefly introduce GPR [55]. A Gaussian process (GP) is a collection of random variables, any finite number of which has a joint Gaussian distribution. A GP is completely specified by its mean function m(x) and covariance function k(x, x’). A GP is denoted as:

(1) f(x) ~GP (m(x), k(x,x’))

Usually, for notational simplicity, we consider the mean function to be zero. 

Let us consider the case where observations $\left\{\left(x_{i},y_{i}\right)|i=1,\ldots ,n\right\}$ are noisy, i.e., y=f(x)+ε, where $\varepsilon \;\sim \;N\left(0,\sigma_{n}^{2}\right)$, assuming additive-independent identically distributed Gaussian noise ε with variance $\sigma_{n}^{2}$. The prior probability on noisy observations becomes:

(2) $$\mathrm{cov}\left(y\right)=K+\sigma_{n}^{2}I.$$

We can write the joint distribution of observed target values and function values at test locations under the prior as:

(3) $$\left[\begin{array}{c} y\\ f_{*} \end{array}\right]\;\sim \;N\left(0,\;\left[\begin{array}{cc} K+\sigma_{n}^{2}I. & K_{*}\\ K_{*} & K_{**} \end{array}\right]\right).$$

The key predictive equation for GPR is:

(4) $$f_{*}|X,y,X_{*}\;\sim \;N\left(\bar{f_{*}},\;\mathrm{cov}\left(f_{*}\right)\right),$$

where:

(5) $$\bar{f_{*}}≜E[f_{*}|X,y,X_{*}]=K_{*}^{T}{\left[K+\sigma_{n}^{2}I\right]}^{-1}y,$$

(6) $$\mathrm{cov}\left(f_{*}\right)=K_{**}-K_{*}^{T}{\left[K+\sigma_{n}^{2}I\right]}^{-1}X_{*}.$$

**Figure 9:**
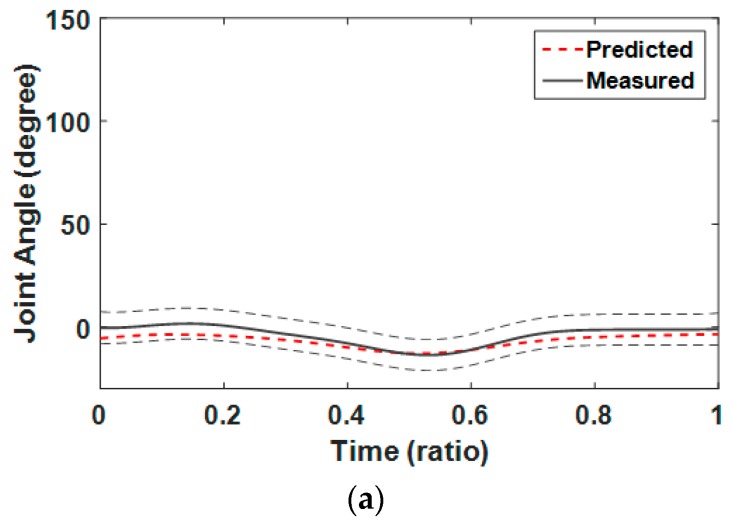

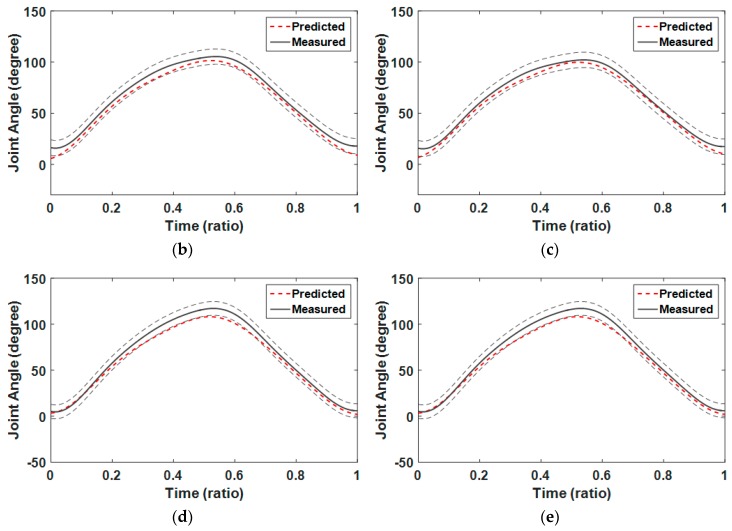
Comparison of squat motion generated using GPR algorithm with average motion determined in the case where foot position was 0.07 h and guide angle was 5° from barbell bar center: flexion/extension of (**a**) pelvis–thorax; (**b**) left hip; (**c**) right hip; (**d**) left knee; and (**e**) right knee.

To validate our motion synthesis algorithm, we compared generated Smith squat motions with measured ones for the five cases marked by the triangular symbol in Table 1. As shown in Figure 9, squat motion generated using the GPR algorithm is compared to average motion determined in the experiment for the case where the foot position was 0.07 h and guide angle was 5° from the barbell bar center. The graphs show that the predicted joint angle patterns were very similar to the measured ones. To analyze the predictive power of the GPR algorithm, we calculated NRMSEs and correlation coefficients between the joint angles of the synthesized squats and those of the measured squats for the four cases marked by the triangular symbol in Table 1. As shown in Table 6, average NRMSEs for pelvis–thorax, hip, and knee joints were 34.6%, 9.2%, and 8.5%, respectively. Total average NRMSE for all joints and cases was 14.0%. Although the average NRMSE for the pelvis-thorax joint was relatively high, it was because the joint angles were very close to zero. NRMSEs show that the GPR-based motion synthesis algorithm can predict real motions quite well. As shown in Table 7, the average correlation coefficients of pelvis–thorax, hip, and knee joints were 0.978, 0.994, and 0.993, respectively. The total average of the correlation coefficients of all joints was 0.988. The high positive correlation coefficients show that the motion synthesis algorithm is very powerful in predicting squat motions.

## 5. Biomechanical Analysis

**Figure 10:**
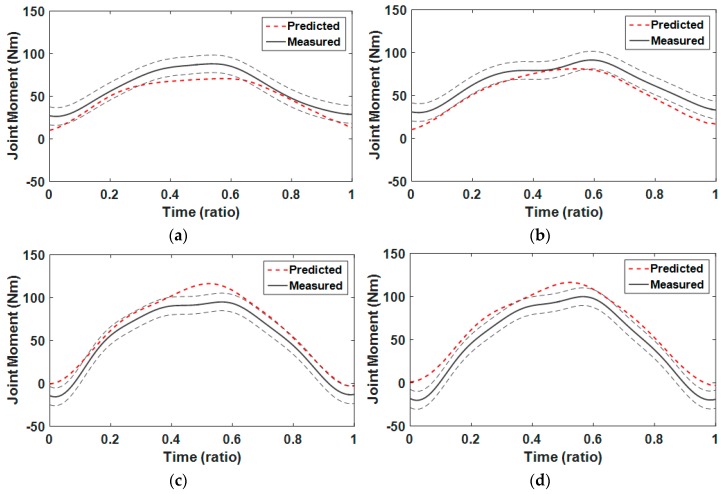
Comparison of moments of hip and knee joints for synthesized squat motion with those for average captured motion in the case where foot position was 0.07 h and guide angle was 5° from barbell bar center: (**a**) left hip moment; (**b**) right hip moment; (**c**) left knee moment; and (**d**) right knee moment.

Biomechanical analyses of Smith squat movements were performed using the integrated human–machine model via AnyBody. To validate synthesized motions, their joint moments were simulated and compared with those of captured motions. For the four cases marked by the triangular symbol in Table 1, squat motions synthesized by the GPR algorithm were analyzed and their results were compared with those of captured motions. As shown in Figure 10, the moments of hip and knee joints for synthesized squat motion are compared with those for average captured motion in the case where the foot position was 0.07 h hand the guide angle was 5° from the barbell bar center. The graphs show that the predicted joint moment patterns were very close to the measured ones. The similarity of joint moments of these two motions was statistically analyzed using NRMSE and Pearson correlation coefficients, as shown in Table 8 and Table 9. Average NRMSEs for the left hip and knee joints were 23.4% and 30.2%, respectively, and those for the right hip and knee joints were 15.6% and 17.4%, respectively. Total average NRMSEs for all joints and cases was 21.7%. As shown in Table 9, the correlation coefficients of pelvis–thorax, hip, and knee joints were higher than 0.97, and their total average was 0.984.

## 6. Analysis Results and Discussion

**Figure 11:**
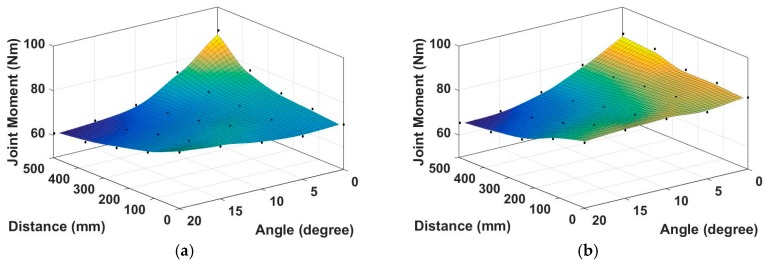

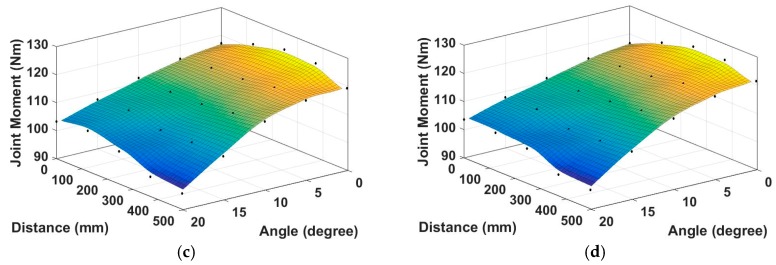
Simulated maximum moments of hip and knee joints for synthesized motions: (**a**) left hip moments and (**b**) right hip moments. (**c**) left knee moments and (**d**) right knee moments.

We selected a subject with 1770 mm of height and performed biomechanical analysis for the 25 cases with different foot positions ranging from 0 to 0.28 h in 0.07 h intervals and various guide angles ranging from 0° to 20° in 5° intervals from the barbell bar center. Squat motions for the 25 cases were generated using the GRP-based motion synthesis algorithm. All joint moments and muscle activities of the model were evaluated for synthesized motions. In addition, joint moments and muscle activities were evaluated for nine captured motions to compare the analysis results of synthesized motions. Figure 11 displays the maximum moments of the right hip and knee joints for synthesized and captured motions. Actually, the signs of the hip and knee moments were negative in AnyBody. However, as the signs are dependent on the choice of coordinate systems, we plotted the magnitudes of the moments in the graphs.

**Figure 12:**
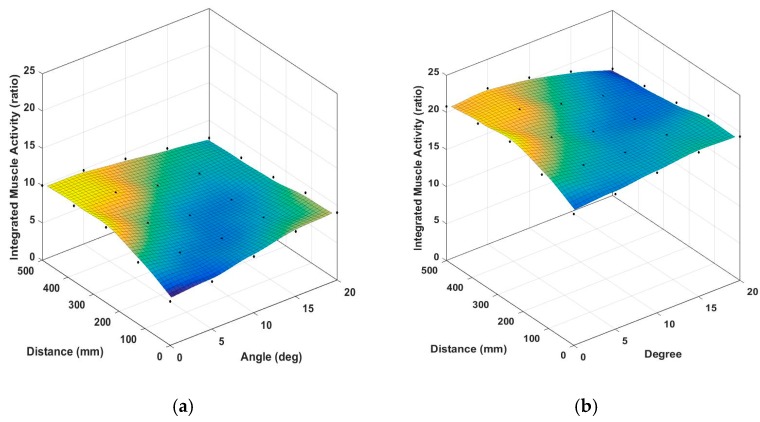

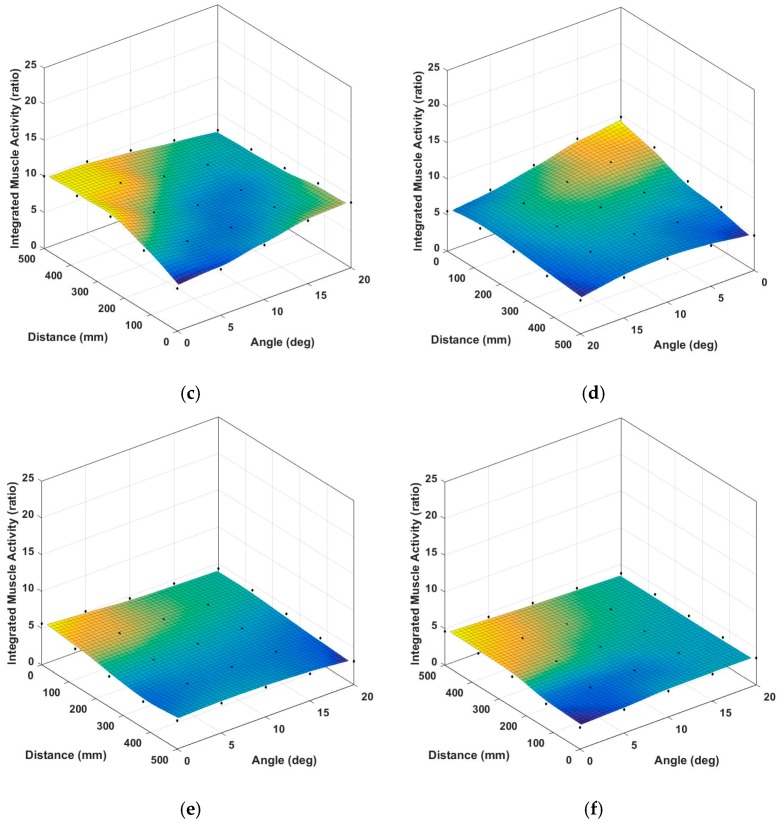
Average muscle activities of right leg simulated for synthesized motions (units: no unit): (**a**) rectus femoris; (**b**) vastus lateralis; (**c**) vastus medialis; (**d**) semitendinosus; (**e**) adductor longus; (**f**) gluteus maximus.

As shown in Figure 11a, the moments of the right and left hip joints were maximum in the case where the foot position (D) was 0.28 h (=500 mm) and guide angle (θ) was 0° and minimum when D = 0.28 h and θ = 20° from the barbell bar center. The right hip moment decreased as D and θ increased. As shown in Figure 11b, the moments of the right knee joint were maximum when D = 0.21 h and θ = 0° and minimum when D = 0.28h and θ = 20° from the barbell bar center. The moments of the right knee decreased as D and θ increased. The average muscle activities of the quadriceps of the right leg during a cycle of squat motion were simulated and plotted for the rectus femoris and vastus lateralis in Figure 12a,b. The patterns of the other quadriceps muscles such as vastus intermedius and vastus medialis are similar to that of the vastus lateralis as illustrated in Figure 12c. The muscle activity increased as D increased in the range of 0° ≤ θ < 15° and decreased as D increased in the range of 15° ≤ θ < 20°. The muscle activity decreased as θ increased. Exceptionally, the activity increased in the range of 0° ≤ θ < 5° and 0.14 h (=250 mm) ≤ D ≤ 0.28 h (=500 mm). The muscle activities of the hamstrings such as the bicep femoris and semitendinosus showed some different pattern from the quadricepts as plotted in Figure 12d. The average muscle activities of the gluteus maximus of the right leg increased as D increased and decreased as θ increased as shown in Figure 12d.

As mentioned in Section 2.1, in this study, only two dominant independent variables, i.e., the foot position and slope of the barbell bar guide, were selected from various possible variables. That is, we fixed other variables such as the amount of knee flexion (semi-, half, parallel, and deep squatting); stance width (narrow/wide); foot angle position (adduction/abduction and inversion/eversion); external load type and positioning (bodyweight squat, dumbbell squat, and front/back barbell squat); speed of execution (body-building/dynamic squat); and external load intensity (typically expressed as a percentage of body weight) to be constant. In particular, the participant’s age, sex, and anthropometric parameters were not selected as independent variables. However, by defining the foot position as a function of the participant’s height, the experiment and analysis results would be used for all the range of heights. Although the other variables were omitted in this study, the multivariate Gaussian process regression model used for motion synthesis can be easily expanded by adding these variables as features of the model and trained with experimental data as the training dataset. That is, the framework and process proposed in this study are effective even if the number of variables is increased. The expansion of the variables remains as future work.

## 7. Conclusions

In this paper, we have proposed a 3D virtual test framework and process for the Smith squat exercise on the basis of a GPR-based motion synthesis algorithm and biomechanical analysis system. In the process, a digital human–machine–environment-integrated model is created, in which interactions between a human body and machine or the ground are modeled as joints with constraints at contact points. Smith squat motion is generated using the motion synthesis program with a set of given values for independent variables. Then, the biomechanical analysis system simulates joint moments and muscle activities from the input of the integrated model and squat motion. The analysis results can be utilized for the design of training programs or Smith machines.

Currently, the prototype system has several limitations. First, we considered only two independent variables. The expansion of independent variables remains as future work. If other variables, particularly a user’s age, sex, and anthropometric parameters, are included, the system can be very useful not only in customizing training programs for a specific user but also for optimizing the design parameters of the Smith machine. Fortunately, the GPR model used for motion synthesis can be easily expanded and trained for additional features, and the musculoskeletal model is scalable to sizes of different individuals. To make the system more robust and precise than the current one, it is necessary to collect more training data and vary independent variables over a wide range than those of this study. Furthermore, significant research efforts are required to enhance the precision of biomechanical analysis results by developing a more precise ground force prediction method and more realistic interaction modeling method than those of this study. All these remain as future work.

## Figures and Tables

**Table 1:** Experimental planning for Smith squats (○: 10 trials, △: 3 trials).

Foot Position (d)	Slope of Bar Guides (θ)				
	0°	5°	10°	15°	20°
0	○		○		○
0.07 h		**△**		**△**	
0.14 h	○		○		○
0.21 h		**△**		**△**	
0.28 h	○		○		○

**Table 2:** NRMSEs of measured and simulated muscle activities of right leg.

Independent Variables		Normalized Root Mean Square Errors of Muscle Activities (%)		
Foot Position (Ratio to Height)	Guide Slope (Degree)	Vastus Lateralis	Vastus Medialis	Rectus Femoris
0	0°	23.1	16.9	28.1
0.14 h	0°	17.1	15.6	11.3
0.28 h	0°	21.7	13.6	10.7
0	10°	23.1	14.6	10.2
0.14 h	10°	23.9	15.1	11
0.28 h	10°	23.8	14.9	11.7

**Table 3:** Pearson correlation coefficients of measured and simulated muscle activities of right leg (*: *p* < 0.001).

Independent Variables		Pearson Correlation Coefficients of Muscle Activities		
Foot Position (Ratio to Height)	Guide Slope (Degree)	Vastus Lateralis	Vastus Medialis	Rectus Femoris
0	0°	0.759 *	0.901 *	0.788 *
0.14 h	0°	0.878 *	0.908 *	0.950 *
0.28 h	0°	0.638 *	0.628 *	0.831 *
0	10°	0.915 *	0.950 *	0.966 *
0.14 h	10°	0.893 *	0.933 *	0.970 *
0.28 h	10°	0.838 *	0.838 *	0.878 *

**Table 4:** Peak timing of maximum muscle activates of right leg measured by EMG sensors and simulated by AnyBody.

Independent Variables		Pearson Correlation Coefficients of Muscle Activities					
Foot Position (Ratio to Height)	Guide Slope (Degree)	Vastus Lateralis		Vastus Medialis		Rectus Femoris	
		EMG	Model	EMG	Model	EMG	Model
0	0°	0.62	0.55	0.60	0.53	0.62	0.53
0.14 h	0°	0.69	0.55	0.56	0.55	0.59	0.54
0.28 h	0°	0.67	0.50	0.65	0.50	0.62	0.50
0	10°	0.57	0.54	0.53	0.54	0.57	0.53
0.14 h	10°	0.59	0.55	0.59	0.55	0.53	0.54
0.28 h	10°	0.61	0.55	0.61	0.55	0.61	0.55

**Table 5:** Total muscle activities of right leg measured by EMG sensors and simulated by AnyBody.

Independent Variables		Total Muscle Activities of Muscle Activities					
Foot Position (Ratio to Height)	Guide Slope (Degree)	Vastus Lateralis		Vastus Medialis		Rectus Femoris	
		EMG	Model	EMG	Model	EMG	Model
0	0°	44.5%	45.5%	42.6%	45.0%	40.9%	28.6%
0.14 h	0°	54.5%	52.3%	52.2%	51.7%	47.4%	47.5%
0.28 h	0°	33.4%	50.9%	38.1%	50.3%	34.8%	50.0%
0	10°	35.2%	47.2%	36.1%	46.5%	36.4%	39.9%
0.14 h	10°	44.9%	51.1%	44.2%	50.6%	41.0%	46.4%
0.28 h	10°	42.9%	55.5%	37.4%	50.6%	33.7%	56.3%

**Table 6:** NRMSEs of captured and synthesized joint angles.

Independent Variables		Normalized Root Mean Square Error of Joint Flexion/Extension (%)				
Foot Position (mm)	Guide Slope (°)	Left Hip	Left Knee	Pelvis–Thorax	Right Hip	Right Knee
0.07 h	5	5.6	4.5	21.1	5.4	3.8
0.07 h	15	8.4	7.6	26.8	7.5	7.4
0.21 h	5	13.9	13.0	45.8	7.8	7.1
0.21 h	15	17.5	17.0	44.8	7.4	7.4

**Table 7:** Pearson correlation coefficients of captured and synthesized joint angles (*: *p* < 0.001).

Independent Variables		Pearson Correlation Coefficient (r) of Joint Flexion/Extension				
Foot Position (mm)	Guide Slope (°)	Left Hip	Left Knee	Pelvis–Thorax	Right Hip	Right Knee
0.07 h	5	0.999 *	0.999 *	0.983 *	0.999 *	0.999 *
0.07 h	15	0.994 *	0.993 *	0.975 *	0.997 *	0.997 *
0.21 h	5	0.984 *	0.984 *	0.974 *	0.984 *	0.984 *
0.21 h	15	0.994 *	0.993 *	0.978 *	0.996 *	0.995 *

**Table 8:** NRMSEs of measured and simulated joint moments.

Independent Variables		Normalized Root Mean Square Error of Joint Moments (%)			
Foot Position (mm)	Guide Slope (°)	Left Hip	Right Hip	Left Knee	Right Knee
0.07 h	5	17.8	19.9	11.8	13.4
0.07 h	15	18.5	23.0	14.1	18.5
0.21 h	5	26.0	30.4	21.1	18.6
0.21 h	15	31.1	47.5	15.6	19.3

**Table 9:** Pearson correlation coefficients of measured and simulated joint moments (*: *p* < 0.001).

Independent Variables		Pearson Correlation Coefficient (r) of Joint Moments			
Foot Position (mm)	Guide Slope (°)	Left Hip	Right Hip	Left Knee	Right Knee
0.07 h	5	0.973 *	0.985 *	0.993 *	0.997 *
0.07 h	15	0.980 *	0.978 *	0.988 *	0.989 *
0.21 h	5	0.978 *	0.988 *	0.974 *	0.983 *
0.21 h	15	0.984 *	0.980 *	0.986 *	0.988 *
